# Diagnostic Accuracy of Salivary Periodontal Biomarkers Is Reduced in Patients With Crohn's Disease

**DOI:** 10.1002/cre2.70387

**Published:** 2026-06-01

**Authors:** Alisa Toivanen, Nur, Ismo Räisänen, Mervi Gürsoy, Ulvi Kahraman Gürsoy, Timo Sorsa, Jaana Rautava

**Affiliations:** ^1^ Department of Oral and Maxillofacial Diseases University of Helsinki and Helsinki University Hospital Helsinki Finland; ^2^ Department Periodontics, Faculty of Dentistry Universitas Gadjah Mada Yogyakarta Indonesia; ^3^ Department of Periodontology, Institute of Dentistry University of Turku Turku Finland; ^4^ The Wellbeing Services County of Southwest Finland Turku Finland; ^5^ Department of Medicine and Dental Medicine Karolinska Institutet Stockholm Sweden; ^6^ Department of Pathology HUS Diagnostics Helsinki Finland

**Keywords:** biomarkers, Crohn disease, diagnostic accuracy, inflammation mediators, periodontitis, saliva

## Abstract

**Objectives:**

Patients with Crohn's disease (CD) are at increased risk of developing periodontal disease and may benefit from salivary biomarker–based monitoring for early detection and management. Given the previously reported reduced specificity of the salivary aMMP‐8 test in CD, this study aimed to evaluate alternative salivary biomarkers for periodontal disease.

**Materials and methods:**

Stimulated saliva samples were collected from 35 patients with CD and 35 systemically healthy, matched controls, followed by a comprehensive periodontal examination. Salivary concentrations of total MMP‐8 (tMMP‐8), MMP‐9, and PMN elastase were quantified using ELISA.

**Results:**

In the control group, periodontal status significantly influenced the levels of all studied biomarkers, with significant differences observed in PMN elastase (*p* = 0.012), MMP‐9 (*p* = 0.003), and tMMP‐8 (*p* < 0.001). In contrast, within the CD group, only MMP‐9 levels showed significant variation across periodontal categories (*p* = 0.011). Intergroup comparisons revealed that among periodontally healthy individuals, those with CD had significantly higher salivary PMN elastase (*p* = 0.039) and MMP‐9 (*p* = 0.005) concentrations than controls. Furthermore, CD patients with gingivitis exhibited significantly higher tMMP‐8 levels (*p* = 0.035$) compared to the control group. However, no significant differences were observed between the CD and control groups among patients with periodontitis for any of the biomarkers (*p* ≥ 0.246).

**Conclusions:**

The diagnostic accuracy of salivary tMMP‐8 and PMN elastase was reduced in CD patients, likely reflecting altered neutrophil degranulation dynamics. Salivary MMP‐9 may serve as a promising biomarker linking oral and intestinal inflammatory activity.

## 1 Introduction

Crohn's disease (CD) is an inflammatory bowel disease (IBD), characterized by chronic, transmural granulomatous inflammation affecting the gastrointestinal tract. The pathophysiology of CD is multifactorial, involving a complex interplay among genetic, environmental, microbial, and immunological factors (Indriolo et al. 2011; Schütz et al. 2003). Patients with CD are prone to oral diseases, such as periodontitis, caries, and oral mucosal lesions (Papageorgiou et al. 2017; Schütz et al. 2003). This may be due to increased sugar consumption or insufficient oral hygiene (Schütz et al. 2003), but there may be a link between CD and periodontitis (Imai et al. 2021; Zhang et al. 2021). Similar to IBD, the pathogenesis of periodontitis involves a combination of genetic predisposition, a dysbiotic microbiota, and an excessive host response (Indriolo et al. 2011; Papageorgiou et al. 2017). Notably, the gingival sulcus shares key immunological and structural features with the intestinal mucosa and is similarly exposed to a high microbial load (Jockel‐Schneider et al. 2016). Disruption of the mucosal barrier is a hallmark of both diseases, allowing direct contact between microbial agents and immunocompetent tissue in both the gut and the periodontal pocket (Indriolo et al. 2011).

A central component of CD pathogenesis is believed to be an aberrant immune response in genetically susceptible individuals (Indriolo et al. 2011). Neutrophils exhibit dysregulated function, contributing to persistent inflammation (Ling et al. 2015; Scott and Krauss 2011; Vitkov et al. 2021). Circulating neutrophils in IBD patients display enhanced activation (Koelink et al. 2014). Despite this hyperactivity, tissue injury in CD—especially in regions such as the rectum, ileum, and skin—is paradoxically associated with impaired neutrophil recruitment and reduced production of proinflammatory cytokines such as interleukin‐8 (IL‐8) and interleukin‐1β (IL‐1β) (Marks et al. 2006). Furthermore, elevated levels of matrix metalloproteinase‐8 (MMP‐8) and MMP‐9 have been detected in the intestinal tissues of IBD patients, implicating neutrophil‐driven inflammation in disease progression (Koelink et al. 2014).

Similarly, a key contributor to excessive immune response and periodontal destruction is the presence of hyper‐reactive neutrophils (Indriolo et al. 2011; Lira‐Junior and Figueredo 2016). One of the critical mediators in this process is MMP‐8, also known as neutrophil collagenase (Luchian et al. 2022). It is a collagen‐degrading enzyme stored in specific granules of neutrophils and activated upon degranulation (Schmidt et al. 2018). MMP‐8 has been strongly linked to the progression of periodontitis and is considered one of the key biomarkers of active disease (Kinane 2000; Luchian et al. 2022; Schmidt et al. 2018). Within the periodontium, MMP‐8 plays a critical role in promoting neutrophil granulocyte migration from the circulation to the periodontal sulcus through proteolytic degradation of extracellular matrix and also modifying immune responses by processing non‐matrix bioactive inflammatory mediators (Luchian et al. 2022; Özçaka et al. 2011). Smoking is a well‐recognized environmental risk factor for periodontitis, enhancing neutrophil‐mediated inflammation by stimulating neutrophil degranulation and promoting the release of proinflammatory mediators (Indriolo et al. 2011; Luchian et al. 2022).

MMP‐9, also known as gelatinase B or 92 kDa gelatinase, is a neutrophil subgranule broad‐spectrum proteolytic enzyme. It is one of the most frequently detected mediators in individuals with periodontitis, and elevated expression levels have been proposed as a potential predictive marker for the disease. Within the periodontium, polymorphonuclear neutrophils are the primary source of MMP‐9. The enzyme plays a central role in degrading multiple connective tissue components and is often markedly overexpressed in chronic periodontitis (Luchian et al. 2022). Beyond the oral environment, MMP‐9 is also a key mediator of intestinal epithelial barrier disruption in the progression of IBD, with serum levels positively correlating with the Pediatric Crohn's Disease Activity Index (Kofla‐Dlubacz et al. 2012).

Similar to MMPs, PMN elastase can degrade the extracellular matrix. In addition, it activates MMP‐8 and MMP‐9, thereby contributing to the enzymatic imbalance observed in IBD and potentiating destructive MMP cascades (Wéra et al. 2016; Özçaka et al. 2011). CD patients with active inflammation exhibit significantly higher plasma and fecal levels of PMN elastase compared to those with inactive inflammation (Andus et al. 1993; Langhorst et al. 2016).

A theory suggests that the chronic inflammatory burden associated with IBD causes increased immunological reactivity. This may result in increased release and activation of collagenolytic MMP‐8 in the periodontal tissues of IBD patients, contributing to a greater susceptibility to periodontal tissue‐destructive inflammation and more pronounced clinical signs of periodontitis (Schmidt et al. 2018). Persistent and amplified systemic inflammation involving both MMPs and their regulator, PMN elastase, may underlie the link between these local and systemic inflammatory conditions (Özçaka et al. 2011).

Given the involvement of these molecular mediators, traditional clinical assessments may not fully capture the dynamic nature of the disease. Periodontal status is traditionally evaluated through clinical indices, such as clinical attachment loss (CAL), probing pocket depth (PPD), and bleeding on probing (BOP), supplemented by radiographic analysis of alveolar bone levels. While these methods remain the gold standard, they are primarily retrospective, quantifying past tissue destruction rather than current activity, and are often labor‐intensive and examiner‐dependent. To overcome these limitations, focus has shifted toward biomarkers—objective, measurable indicators of pathogenic shifts and the complex interplay between the subgingival microbiome and the host's inflammatory response. Saliva, an accessible and non‐invasively collected biofluid containing biomarkers from both local and systemic sources, provides a promising platform for high‐sensitivity screening and next‐generation diagnostic tools (Gul et al. 2020; Sha et al. 2024).

According to our previous results (Rautava et al. 2020), we hypothesized that periodontitis marker levels differ in CD patients. In this study, we investigate the salivary concentrations of biomarkers indicative of periodontitis in CD patients. Examining these biomarkers can improve our understanding of how CD affects immune defense mechanisms in the oral cavity, potentially shedding light on the shared pathophysiological background of oral diseases and CD. Additionally, this research may help identify biomarkers that could serve as indicators of oral or intestinal health, thereby aiding in the screening of CD patients who may require special monitoring or treatment.

## 2 Materials and Methods

### 2.1 Study Design and Enrolment of Participants

This study was approved by the Ethical Committee of the Hospital District of Southwest Finland (114/1801/2016). Participants were informed about the study purpose, as well as any potential risks and benefits, before providing written consent.

**Figure 1:**
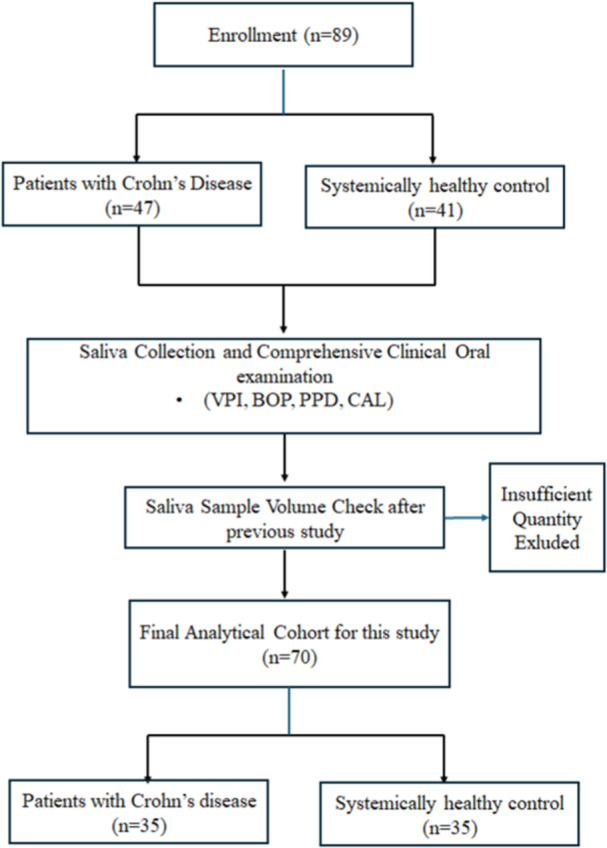
Flowchart of the study population. From the initial cohort (*n* = 89), 70 participants (35 with Crohn's disease and 35 systemically healthy controls) were included in the final analytical sample. Exclusion was based on insufficient saliva volume remaining after previous analyses.

This cross‐sectional study utilized saliva samples from a cohort (*n* = 89) originally collected at the University of Turku in 2017–2018 and described by Rautava et al. (2020). The present study was designed as a pilot investigation and a follow‐up to this previous work, aiming to extend the analysis to novel salivary biomarkers. Due to limited residual sample volumes, a sub‐cohort of 70 participants (35 with CD and 35 healthy controls) was selected for the present analysis. The selection process is detailed in Figure 1.

CD was diagnosed based on endoscopy and histopathological analysis of intestinal biopsy. The control group consisted of individuals without CD or any other autoimmune diseases, matched for age and gender. Exclusion criteria for both groups included diabetes mellitus, smoking, excessive alcohol consumption, having fewer than 24 teeth, use of antimicrobials within the past 6 months, pregnancy, and breastfeeding.

Background information, including age, gender, place of residence, general health, medications, and previous dental treatments, was collected via a questionnaire. The Crohn's Disease Activity Index (CDAI) was used to assess disease activity in CD patients (Best et al. 1976).

### 2.2 Salivary Sampling and Clinical Examination

The study participants were instructed to avoid tooth brushing, eating, and chewing gum 30 min prior to the visit. Paraffin‐stimulated salivary sampling collected for 5 min was performed before the clinical oral examination during afternoon hours (Navazesh 1993). All samples were aliquoted and stored as whole saliva at −70°C until use.

Participants underwent a full‐mouth clinical examination, which has been described in detail previously (Rautava et al. 2020). In brief, mucosal assessments were performed by a single oral pathologist (J.R.), while the rest of the oral examination was completed by a calibrated periodontist (M.G.). Prior to the study, intra‐examiner reliability for the clinical investigator (M.G.) was assessed through duplicate measurements of 10 patients, demonstrating excellent consistency. Periodontal status included measurements of visible plaque index (VPI), BOP, PPD, and clinical attachment level (CAL) at six sites per each natural tooth and dental implant. Additionally, when present, tooth mobility and furcation defects were recorded.

The periodontal diagnosis was determined based on the 2018 Periodontal Disease Classification. Periodontal health was defined as BOP < 15%, PPD < 4 mm, and no CAL loss. Gingivitis was diagnosed when BOP ≥ 15% was present in the absence of PPD ≥ 4 mm or CAL loss. Periodontitis was defined by the presence of PPD ≥ 4 mm and/or interdental CAL loss ≥ 2 non‐adjacent teeth associated with BOP. Disease severity was categorized as follows: Initial periodontitis (PPD = 4 mm, CAL loss 1–2 mm), moderate periodontitis (PPD 5–6 mm, CAL loss 3–4 mm), and severe periodontitis (PPD ≥ 7 mm, CAL loss ≥ 5 mm) (Papapanou et al. 2018).

### 2.3 Measurement of MMM‐9, tMMP‐8, and PMN Elastase

Salivary MMP‐9, tMMP‐8, and PMN elastase concentrations were measured using commercially available enzyme‐linked immunosorbent assay (ELISA) kits: the Human MMP‐9 ELISA Kit and Human PMN Elastase ELISA Kit (Invitrogen, ThermoFisher), and the Human Total MMP‐8 ELISA Kit—Quantikine (R&D Systems, Minneapolis, MN). Assays were performed in duplicate, following the manufacturer's instructions. The saliva samples were pre‐diluted with the provided sample diluent at a ratio of 1:100 for PMN elastase and MMP‐9, and 1:10 for tMMP‐8. The differing dilution ratios were determined based on the expected physiological concentrations of each biomarker to ensure they fell within the assay's optimal detection range. Finally, the samples were analyzed using a spectrophotometric microplate reader (Perkin Elmer Victor X2 Multimode Microplate Plate Reader 2030). The final concentrations were calculated based on a standard curve using Microsoft Excel.

### 2.4 Statistical Analysis

All statistical analyses were performed using the SPSS statistical program (version 24.0; IBM Inc., Armonk, NY, USA). Age, salivary flow rate, and number of teeth were compared between the CD group and control group with one‐way ANOVA test. The Chi‐Square test was used for comparing other demographic and clinical data (except for age and number of teeth) between the groups. Relationships between MMP‐9, tMMP‐8, and PMN elastase concentrations, periodontal disease status, and CD were assessed using the Kruskal–Wallis test, the Dunn–Bonferroni test for pairwise post‐hoc comparisons, and the Mann–Whitney *U* test. Statistical significance was set at *p* < 0.05.

## 3 Results

### 3.1 Characterization of the Study Groups

This study included 35 saliva samples from CD patients and 35 samples from control participants (Table 1). There were 71.4% men in the CD group and 65.7% in the control group. The mean age was 48.1 years (range 22–70 years) and 46.3 years (range 23–72 years), respectively. Among the CD patients, 48.6% had the disease in remission (short CDAI < 150 points), 40.0% had mild disease (short CDAI 150–219 points), 11.4% had moderate disease (short CDAI 220–450 points), and none had severe disease (short CDAI > 450 points). Of the CD patients, 88.6% received medication for CD, and 37.1% had undergone surgical treatment.

**Table 1:** Demographic characteristics and periodontal status of the study groups.

Findings	CD group, *n* = 35	Control group, *n* = 35	*p*‐value
Gender (male %)	71.4	65.7	0.797
Age, years (mean, st. dev)	48.1 (13.3)	46.3 (13.0)	0.575
No. of teeth (mean, st. dev)	26.8 (2.5)	28.0 (2.1)	0.042
BOP % (mean, st. dev)	33.6 (23.8)	30.5 (27.4)	0.604
I Periodontally healthy (*n*, %)	9 (25.7)	15 (42.9)	0.026
II Gingivitis (*n*, %)	13 (37.1)	12 (34.3)	
III Initial periodontitis (*n*, %)	12 (34.3)	3 (8.6)	
IV Moderate periodontitis (*n*, %)	1 (2.9)	2 (5.7)	
V Severe periodontitis (*n*, %)	0 (0)	3 (8.6)	

Abbreviation: BOP = bleeding on probing.

The participants were divided into five clinical periodontal groups: Healthy (I), gingivitis (II), initial periodontitis (III), moderate periodontitis (IV), and severe periodontitis (V). Based on periodontal status, 25.7% of the CD group were classified as group I, 37.1% as group II, 34.3% as group III, and 2.9% as group IV. No participants in the CD group were classified in group V. In the control group, 42.9% were classified as group I, 34.3% as group II, 8.6% as group III, 5.7% as group IV, and 8.6% as group V. The numbers of individuals in periodontal groups IV and V were insufficient for reliable statistical analysis; therefore, the analyses were restricted to groups I–III.

In the CD group (*n* = 35), 62.9% (*n* = 22) were receiving active pharmacological treatment for their condition, while 37.1% (*n* = 13) were not on any medication. Among those treated, 13 patients (37.1%) were on monotherapy, and 9 patients (25.7%) were receiving combination therapy. The specific medications included 5‐aminosalicylates (5‐ASA; *n* = 14, consisting of 12 patients on mesalazine and 2 on sulfasalazine) and immunosuppressive agents (*n* = 15, consisting of 9 patients on azathioprine, 5 on mercaptopurine, and 1 on methotrexate). Biological therapy (infliximab) was used by 2 patients, and 1 patient was receiving corticosteroid treatment (budesonide). Due to the sample size and the further division of patients into five different periodontal health categories, the cohort was not stratified by medication type for the primary statistical analysis.

### 3.2 PMN Elastase Concentrations in Periodontal Diseases and Crohn's Disease Groups

In the CD group, no significant difference in PMN elastase levels (*p* = 0.118, Kruskal–Wallis test) was observed between the various periodontal groups (Figure 1). However, in the control group, significant variation in PMN elastase levels was observed (*p* = 0.012, Kruskal–Wallis test). Within the control group, a clinical diagnosis of periodontitis was characterized by significantly higher salivary PMN elastase concentrations (*p* = 0.015 compared to Group I, *p* = 0.035 compared to Group II, Dunn–Bonferroni test).

**Figure 2:**
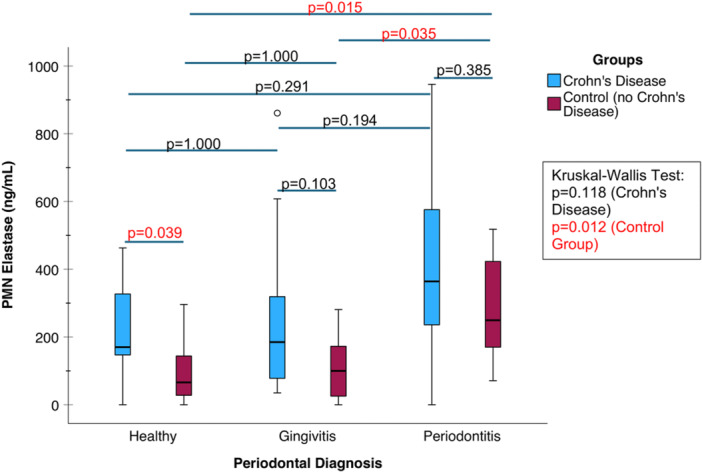
Comparison of PMN elastase concentrations between the CD and control groups using box plots.

Among individuals with a healthy periodontium, salivary PMN elastase levels (*p* = 0.039, Mann–Whitney *U* test) were higher in the CD group compared to the control group. In contrast, no significant difference in PMN elastase levels was found between the CD and control groups among periodontitis patients (*p* = 0.385, Mann–Whitney *U* test) (Figure 2).

### 3.3 MMP‐9 With Concentrations in Periodontal Diseases and Crohn's Disease Groups

**Figure 3:**
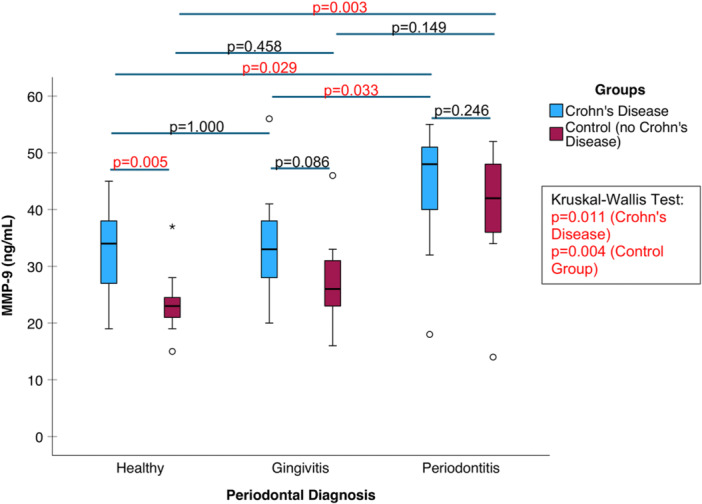
Comparison of MMP‐9 concentrations between the Crohn's disease and control groups using box plots.

In the CD group, significant variation in MMP‐9 levels (*p* = 0.011, Kruskal–Wallis test) was observed between the various periodontal groups (Figure 3). Specifically, the CD group with periodontitis (Group III) showed significantly elevated MMP‐9 levels compared to both Group I (*p* = 0.029, Dunn–Bonferroni test) and Group II (*p* = 0.033, Dunn–Bonferroni test). Similarly, in the healthy control group, periodontitis (Group III) was linked to significantly higher salivary MMP‐9 concentrations (*p* = 0.003 compared to Group I, no significant difference compared to Group II, Dunn–Bonferroni test). No significant difference in MMP‐9 levels was observed between the CD and control groups in individuals with periodontitis (*p* = 0.246, Mann–Whitney test). In individuals without periodontal disease, the CD group had higher salivary concentrations of MMP‐9 (*p* = 0.005, Mann–Whitney *U* test).

### 3.4 tMMP‐8 Concentrations in Periodontal Diseases and Crohn's Disease Groups

**Figure 4:**
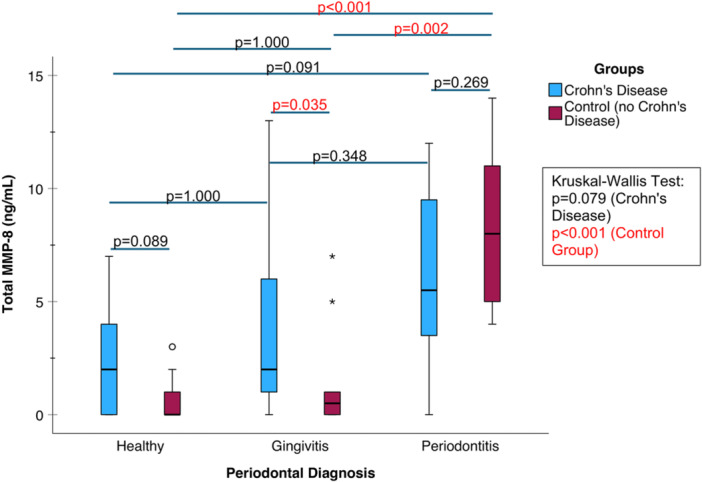
Comparison of tMMP‐8 concentrations between the Crohn's disease and control groups using box plots.

In the CD group, no significant differences were found in tMMP‐8 concentrations (*p* = 0.079, Kruskal–Wallis test) between the different periodontal groups (Figure 4). However, individuals with a clinical diagnosis of gingivitis in the CD group had higher tMMP‐8 levels (*p* = 0.035, Mann–Whitney *U* test) compared to the control group.

In the healthy control group, a clinical diagnosis of periodontitis corresponded to significantly higher salivary tMMP‐8 concentrations (*p* < 0.001 compared to Group I, *p* = 0.002 compared to Group II, Dunn–Bonferroni test). No significant difference in tMMP‐8 levels was observed between the CD and control groups in periodontitis patients (*p* = 0.269, Mann–Whitney *U* test).

## 4 Discussion

In this study, we observed differential salivary biomarker levels between individuals with CD and controls. Notably, elevated salivary MMP‐9 concentrations were significantly linked to periodontitis within the CD cohort. This finding suggests a potential role for salivary MMP‐9 as a minimally invasive marker that may reflect both oral and gastrointestinal inflammation. While these preliminary results are encouraging, further validation in larger cohorts is needed before its utility in the screening of CD patients requiring specialized monitoring can be fully established.

In CD, the diagnostic utility of salivary tMMP‐8 as an indicator of periodontitis is attenuated, consistent with our earlier findings indicating a similarly reduced diagnostic performance for active MMP‐8 (aMMP‐8) measurements in oral rinse samples (Rautava et al. 2020). This supports the concept that tMMP‐8, that is, pro‐ or latent collagenolytically inactive MMP‐8, evidently acts as a storage and/or source of active collagenolytic MMP‐8 (Luchian et al. 2022; Schmidt et al. 2018; Özçaka et al. 2011). Activation cascade of tMMP‐8 is seemingly, at least in part, defective in CD.

In our study within the CD group, PMN elastase levels did not show significant differences across periodontal health statuses. Among individuals with a healthy periodontium, salivary PMN elastase levels were significantly elevated in the CD group compared to controls. This aligns with prior studies demonstrating that increased PMN elastase levels in plasma and fecal samples reflect gastrointestinal disease activity and severity (Andus et al. 1993; Barry et al. 2020). Consequently, further investigation is warranted to determine whether salivary PMN elastase can serve as a reliable biomarker for assessing CD severity.

The role of MMP‐9 in the oral health of CD patients was evaluated for the first time to our knowledge. Within the CD group, significant differences in MMP‐9 salivary levels were observed according to periodontal health: Individuals with periodontitis exhibited markedly higher MMP‐9 concentrations. However, when comparing periodontitis patients across the CD and control groups, no significant difference in MMP‐9 levels was found. Among those without periodontal disease, the CD group displayed elevated salivary MMP‐9 levels. This may reflect the phenomenon that MMP‐9 is often overexpressed in the inflamed intestinal tissue of CD and plays a primary role in mediating tissue degradation in the gut. This is based on present results, also concerning the oral cavity, which is a part of the gastrointestinal tract.

Notably, the CD group exhibited fewer periodontally healthy individuals, a higher prevalence of gingivitis and mild periodontitis, and a greater average number of missing teeth—findings consistent with existing literature on impaired oral health in CD (Papageorgiou et al. 2017). Conversely, while previous studies have reported greater prevalence and severity of periodontitis in CD (Papageorgiou et al. 2017), in our cohort, severe periodontitis was observed solely in the control group. This discrepancy may stem, at least in part, from the rather limited sample size, which is a challenge due to the relative rarity of CD in the Finnish population. Additionally, the cross‐sectional design of this study precludes any inference of causality.

The use of saliva as a diagnostic medium has become well‐accepted. It contains a diverse array of biomarkers, and its non‐invasive collection is simple, cost‐effective, and free from specialized equipment needs, making it well‐suited for screening large populations. These characteristics are particularly advantageous for disease screening and longitudinal monitoring. However, a key limitation is the inherently low biomarker concentrations in saliva, necessitating highly sensitive analytical techniques such as ELISA. Biomarker stability also relies on stringent storage conditions (e.g., −80°C). Furthermore, saliva composition shows substantial variability influenced by individual health, age, and environmental factors such as time of day, dietary intake, oral hygiene, and physical activity. In this study, we employed standardized collection protocols to ensure sample comparability; nevertheless, the lack of universally accepted saliva collection standards continues to hamper cross‐study reproducibility (Liu and Duan 2012).

A major strength of this study is the high‐quality patient cohort: CD was diagnosed through both endoscopic and histopathological means, and disease activity was quantified using the Crohn's Disease Activity Index (CDAI). Mucosal examinations were performed by a single oral pathologist, as well as a single periodontist assessed both cariological and periodontal status, minimizing inter‐observer variability. The study's validity is further supported by the absence of statistically significant differences between the CD and control groups in sex and age.

The present study has several limitations. First, as it was conducted as a pilot study, the sample size was limited. In addition, all samples were collected from a single research unit in the city of Turku; therefore, the study cohort may not be fully representative of the general population. Second, the patient and control cohorts were not matched for BMI. This is noteworthy, as evidence from a separate study has demonstrated that obesity contributes to elevated circulating concentrations of MMP‐8 (Lauhio et al. 2016).

Third, although saliva was used as the oral fluid biomarker matrix in the present study, it should be noted that oral rinse samples—obtained after centrifugation—appear to provide a more accurate oral fluid matrix for biomarker analysis than 5‐min unstimulated saliva samples (Katsiki et al. 2021).

Fourth, significant baseline differences were observed between the CD and control groups regarding the severity of periodontitis and the number of teeth. While adjusting for these potential confounders through multivariate analysis would be ideal, the small sample size of this pilot study precluded reliable regression modeling without the risk of over‐fitting.

Finally, the analysis was constrained by the heterogeneity and the relatively small number of participants receiving specific medical treatments. In the CD group, 22 patients (62.9%) were on active medication, with 13 receiving monotherapy and 9 on combination therapy. The treatments included 5‐ASAs (*n* = 14), immunosuppressants (*n* = 15), corticosteroids (*n* = 1), and biological medications (*n* = 2). Due to this limited subgroup size, we lacked the statistical power to evaluate potential confounding or synergistic effects of these advanced therapies on biomarker levels. Furthermore, medical records for one individual in the control group were unavailable, and thus, medication history could not be verified for this participant. Future studies with larger, more homogeneous or stratified cohorts are warranted to isolate the specific effects of different CD medications on oral health and periodontal biomarkers.

The clinical status of the CD group also warrants consideration, as nearly half of the cohort was in clinical remission. Patients in remission often present with a systemic inflammatory status that parallels that of systemically healthy controls, which may have homogenized the study groups and attenuated differences in biomarker levels. While our categorical classification (Healthy, Gingivitis, Periodontitis) aimed to distinguish oral conditions, the presence of systemically stable CD likely influenced the overall results.

These limitations underscore the necessity for future research to prioritize larger, multicenter cohorts. Such an approach would allow for more granular stratification based on both systemic disease activity and local periodontal stability, ensuring sufficient power to isolate the impact of specific pharmacological interventions on these biomarkers.

While the current study focused on individual biomarkers, investigating biomarker ratios—such as MMPs in relation to their tissue inhibitors (e.g., TIMP‐1)—could provide a more robust reflection of the proteolytic environment in CD. Given the diagnostic potential of the aMMP‐8/TIMP‐1 ratio, this remains a key objective for future research utilizing larger sample volumes.

In conclusion, the diagnostic accuracy of salivary periodontal biomarkers appears to be altered in the presence of CD. In systemically healthy controls, all three biomarkers were significantly linked to periodontitis; however, within the CD cohort, only MMP‐9 levels significantly reflected the periodontal disease process. Notably, MMP‐9 and PMN elastase levels were significantly elevated in CD patients even in the presence of a healthy periodontium. While these findings provide preliminary insights into a shared pathophysiological background between periodontitis and CD, they should not be generalized without further large‐scale validation. Further examination of these biomarkers could improve our understanding of how CD modulates immune defense mechanisms in the oral cavity, which is essential for developing novel therapeutic strategies.

## Author Contributions

**Alisa Toivanen:** writing – original draft, investigation, methodology, visualization, formal analysis, writing – review and editing. **Nur:** methodology, formal analysis, writing – review and editing. **Ismo Räisänen:** visualization, writing – review and editing, data curation, software. **Mervi Gürsoy:** conceptualization, investigation, methodology, validation, writing – review and editing. **Ulvi Kahraman Gürsoy Ulvi Kahraman:** conceptualization, investigation, methodology, writing – review and editing, project administration. **Timo Sorsa:** methodology, writing – review and editing, formal analysis, supervision, resources. **Jaana Rautava:** project administration, conceptualization, investigation, funding acquisition, methodology, writing – review and editing, formal analysis, data curation, supervision, resources.

## Funding

The authors have nothing to report.

## Ethics Statement

This work was approved by the Ethical Committee of the Hospital District of Southwest Finland (114/1801/2016). Written informed consent was obtained from all participants in accordance with the Declaration of Helsinki.

## Conflicts of Interest

Timo Sorsa is the inventor of the following patents relating to aMMP‐8 lateral flow testing: US patents 5,652,223, 5,736,341, 5,866,432, 6,143,476, 2017/0023571A1 (granted 6 June 2019), WO2018/060553A1 (granted 31 May 2018), 10488415B2, and Japanese patent 2016‐554676 and South Korean Patent No. 10–2016‐7025378. Timo Sorsa owns shares at Koite Health Ltd., Berries United Ltd., Orion Ltd., SpectroCare Ltd., and Dentognostics Ltd. The other authors declare no conflicts of interest.

## Data Availability

The data that support the findings of this study are available on request from the corresponding author. The data are not publicly available due to privacy or ethical restrictions.
